# Real-Time Functional Stratification of Tumor Cell Lines Using a Non-Cytotoxic Phospholipoproteomic Platform: A Label-Free Ex Vivo Model

**DOI:** 10.3390/biology14080953

**Published:** 2025-07-28

**Authors:** Ramón Gutiérrez-Sandoval, Francisco Gutiérrez-Castro, Natalia Muñoz-Godoy, Ider Rivadeneira, Adolay Sobarzo, Jordan Iturra, Ignacio Muñoz, Cristián Peña-Vargas, Matías Vidal, Francisco Krakowiak

**Affiliations:** 1 Department of Oncopathology, OGRD Alliance, Lewes, DE 19958, USA; consultorusa@biogenica.org (C.P.-V.); mvidaldominguez.1992@gmail.com (M.V.); 2 Cancer Research Department, Flowinmunocell-Bioexocell Group, 08028 Barcelona, Spain; servicios@flowinmunocell.cl (F.G.-C.); contacto@flowinmunocell.cl (N.M.-G.); 3 Outreach and Engagement Programs Department for the OGRD Consortium, Charlestown KN0802, Saint Kitts and Nevis; iderlautaro@gmail.com (I.R.); jiconsultant@ogrdconsorcio.com (J.I.); kinesiologo@recell.cl (I.M.); 4 Departamento de Ciencias Biológicas y Químicas, Facultad de Ciencias, Universidad San Sebastián, Lientur 1457, Concepción 4080871, Chile; adolay.sobarzo@uss.cl; 5 Department of Molecular Oncopathology, Bioclas, Concepción 4030000, Chile; fkconsultant@ogrdconsorcio.com

**Keywords:** phospholipoproteomic platform, functional tumor stratification, ex vivo immunoprofiling, non-cytotoxic kinetic modeling, immunophenotypic classification, IFN-γ/IL-10 ratio, structural immunomodulation, STIP traceability system, batch-level documentation, early-stage regulatory validation

## Abstract

The development of scalable, non-invasive tools to assess tumor responsiveness to structurally active immunoformulations remains a critical unmet need in solid tumor immunotherapy. Here, we introduce a real-time, ex vivo functional system to classify tumor cell lines exposed to a phospholipoproteomic platform, without relying on cytotoxicity, co-culture systems, or molecular profiling. Tumor cells were monitored using IncuCyte^®^ S3 (Sartorius) real-time imaging under ex vivo neutral conditions. No dendritic cell components or immune co-cultures were used in this mode. All results are derived from direct tumor cell responses to structurally active formulations. Using eight human tumor lines, we captured proliferative behavior, cell death rates, and secretomic profiles to assign each case into stimulatory, inhibitory, or neutral categories. A structured decision-tree logic supported the classification, and a Functional Stratification Index (FSI) was computed to quantify the response magnitude. Inhibitory lines showed early divergence and high IFN-γ/IL-10 ratios; stimulatory ones exhibited a proliferative gain under balanced immune signaling. The results were reproducible across independent batches. This system enables quantitative phenotypic screening under standardized, marker-free conditions and offers an adaptable platform for functional evaluation in immuno-oncology pipelines where traditional cytotoxic endpoints are insufficient. This approach has been codified into the STIP (Structured Traceability and Immunophenotypic Platform), supporting reproducible documentation across tumor models. This platform contributes to upstream validation logic in immuno-oncology workflows and supports early-stage regulatory documentation.

## Simple Summary

Functional profiling of a tumor’s response to non-toxic phospholipoproteomic platforms is a growing field in oncology. Unlike traditional drug models, which rely on killing cells or tracking receptor inhibition, this study presents a real-time, label-free system to classify tumor phenotypes based on their kinetic and secretomic behavior. By avoiding cytotoxic endpoints or genetic manipulation, we captured functional compatibility between human tumors and structurally active phospholipoproteomic formulations. This platform supports the standardized classification of tumor responses into stimulatory, inhibitory, or neutral profiles, providing a reproducible and non-invasive tool for selecting candidates in preclinical cancer immunotherapy programs.

## 1. Introduction

### 1.1. Limitations of Conventional Preclinical Models

Traditional preclinical models used in immuno-oncology—such as IC_50_ assays, tumor regression in mice, or lympho-tumor co-cultures—have structural limitations when evaluating non-pharmacodynamic platforms such as dendritic phospholipoproteomic immunoformulation. These systems typically rely on toxicity or enzyme inhibition measures, which are methodologically inapplicable to products that do not induce cell lethality or interact with defined molecular targets. Furthermore, murine models implanted into immunodeficient animals lack human immunological features, limiting the extrapolation of immune-structural effects induced by phospholipoproteomic formulations.

Added to this is the impossibility of modeling fine-grained topological interactions, such as phospholipoproteomic immunoformulation-tumor structural recognition, which does not respond to pharmacokinetic principles or dose–response curves. Faced with this experimental dilemma, there is a growing need to incorporate systems that can capture real functional responses—such as alterations in proliferative rhythm—without relying on cell destruction or direct immune activation. This need has driven the search for structured kinetic models capable of reflecting phenotypic changes induced by complex immunogenic stimuli under neutral, reproducible, and quantifiable technical conditions [1,2].

In this study, the term phospholipoproteomic platform does not refer to classical vesicles, but rather to an ultrapure, structurally active formulation composed of non-vesicular phospholipid–protein fractions that are organized into a stable proteolipidic architecture. These platforms exhibit reproducible immunostructural properties and are devoid of genetic coding elements or replicative potential. Their action is functional, not cytotoxic, and is interpreted as a phenotypic interface between bioactive structures and tumor cells under neutral ex vivo conditions.

### 1.2. Functional Justification for Real-Time Kinetic Monitoring

The use of automated kinetic capture systems, such as IncuCyte^®^, overcomes many of the technical barriers associated with terminal viability models. Through continuous, real-time monitoring of cell confluence, it is possible to record dynamic events that reflect genuine functional alterations without the need for dyes, fixation, fluorogenic markers, or destructive intervention [3]. This is particularly relevant for immunoactive phospholipoproteomic immunoformulation, whose action is structural, non-cytotoxic, and not mediated by conventional pharmacological mechanisms.

The neutral readout offered by IncuCyte^®^ represents a critical advantage: it allows quantification not only of whether a change in cell proliferation takes place, but also when it occurs, how long it is sustained, and at what magnitude relative to the control. This type of information is essential for interpreting functional responses that do not result in cell death but demonstrate significant phenotypic reprogramming. Furthermore, by eliminating all manual manipulation during the observation period, the system ensures high technical reproducibility with minimal operational interference.

In this context, kinetic monitoring is established as an ideal tool for assessing gallbladder-tumor functional compatibility under controlled conditions, serving as a robust primary readout for functional classifiers without resorting to destructive assays or artificial experimental conditions [3].

### 1.3. Emerging Role of Phospholipoproteomic Immunoformulation in Structural Immunoreprogramming

Ultrapurified phospholipoproteomic platforms have emerged as next-generation immunobiological tools due to their ability to induce phenotypic reprogramming in tumor cells without requiring conventional pharmacological signaling or triggering direct cytotoxic effects. Unlike conventional tumor exosomes or epithelial microplatforms loaded with genomic material, these formulations are enriched with immunoactive molecular patterns—such as MHC I/II, tetraspanins (CD9, CD81), ICAM-1, and structural cytokines—that enable complex functional interactions with target cells, reorganizing their behavior without causing cell lysis or death [4,5].

This unique feature makes phospholipoprotein fractions ideal candidates for structural immunomodulation platforms, where the objective is not to destroy the target tissue, but rather to modify its basal phenotypic dynamics in a controlled manner. However, cellular interpretation of these vesicular stimuli is not uniform: different tumor lines respond divergently to structurally similar formulations, suggesting the existence of a functional axis of immunophenotypic compatibility that has not yet been fully characterized [4].

This compatibility cannot be assessed solely through toxicity or single biomarkers; it requires ex vivo functional models that are capable of reproducibly quantifying the impact of these stimuli on cell proliferative kinetics. In this context, continuous real-time readout using platforms, such as IncuCyte^®^, (Essen BioScience Inc., Ann Arbor, MI, USA; Essen BioScience Inc., Ann Arbor, MI, USA; software version 2019B) applied to structurally validated phospholipoproteomic formulations, allows the degree of tumor–platform functional compatibility to be accurately captured without the need for systemic immunological intervention or immediate clinical validation.

### 1.4. Basis of Platform–Tumor Functional Classification

The central hypothesis underlying the model proposed here is that the interaction between an immunoactive phospholipoprotein vesicular fraction and a human tumor cell line generates differential, quantifiable, and classifiable proliferative behavior under neutral experimental conditions. This interaction is not based on specific molecular affinities or classical pharmacodynamic mechanisms, but rather on the structural compatibility between the immunoactive vesicular content and the basal phenotype of the target cell [5].

Based on the analysis of divergent growth kinetic trajectories, it is possible to establish a robust and reproducible functional classification: (i) stimulated lines, which increase their proliferation steadily after vesicular exposure; (ii) inhibitory lines, which show a progressive reduction in their replication rate without signs of cell death; and (iii) inert lines, whose dynamics do not change significantly in response to the stimulus. This typology has technical and operational value in both research and regulatory settings: it allows objective discrimination between sensitive, resistant, or indifferent models, without the need to know the genetic or transcriptomic profile of each line. Furthermore, real-time quantification allows the system to capture not only the final effect but also the stability, duration, and reproducibility of the response. This structure offers an objective functional tool, which is useful for technical decisions in early validation, immunophenotypic classification, indicator line selection, and defensible functional documentation in the absence of direct clinical evidence [6,7].

### 1.5. Emerging Regulatory Logic: Early Validation and Documentation Platforms

In parallel with scientific progress, various national and international regulatory authorities have begun to incorporate unconventional functional validation schemes, especially in the case of non-cellular, non-pharmacodynamic, and structurally immunomodulatory products, such as phospholipoproteomic formulations like PLPC-DB. PLPC-DB refers to an ultrapure, lyophilized phospholipoproteomic formulation derived from the secretome of human dendritic cells that were previously pulsed with a defined neoantigen algorithm, exhibiting immunomodulatory properties without genetic or pharmacodynamic content.

In these regulatory environments, it is recognized that it may be acceptable to present non-clinical technical evidence as long as it is reproducible, quantifiable, and consistent with the proposed mechanism of action, even in the absence of direct clinical validation. This shift has enabled the development of documentation platforms aimed at supporting early structural phenotypic compatibility by using functional technical tools that do not depend on cell destruction or conventional pharmacodynamic assays [8,9].

Within this framework, ex vivo kinetic models acquire potential regulatory value by allowing the identification of tumor-gallbladder functional trajectories under controlled conditions and without destructive markers or active immunological intervention. This type of evidence is also compatible with regulatory pathways that allow for delayed activation, technical documentary use, or exclusion from classification as a new pharmacological entity, provided that technical traceability, safety, and defensible functional logic are demonstrated. Therefore, the model proposed here not only responds to an experimental need but also offers a concrete opportunity to inform modular technical dossiers (such as the CTD) in contexts where immediate clinical efficacy is not required but rather structured technical consistency is [10].

### 1.6. Objective of the Study and Operational Experimental Framework

The present study aimed to establish and validate a technical functional model that allows the reproducible classification of the proliferative response of human tumor lines exposed to immunoactive phospholipoproteomic formulations under neutral, marker-free ex vivo conditions. The operational hypothesis holds that cell growth kinetics reflects the structural interpretation of the immunoactive stimulus, and that this response can be organized into a functional typology with technical value. To this end, a cohort of eight tumor cells with diverse histological profiles was used, exposed to phospholipoproteomic formulations prepared under controlled conditions, and quantified by protein concentration. Proliferation curves were captured in real time for 48 h using the IncuCyte^®^ platform, complemented by cell death analysis and multiplex secretomic profiling [11].

**Figure 1:**
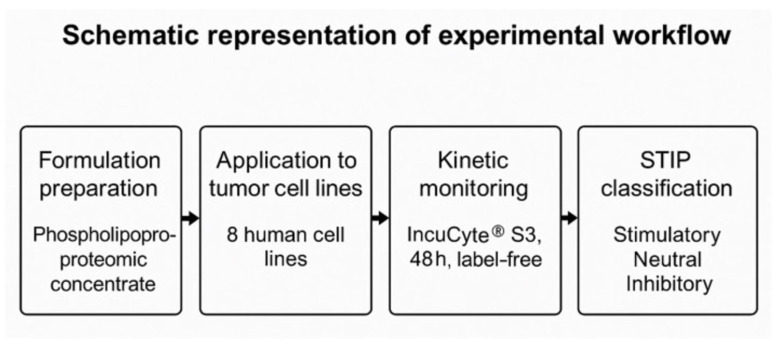
Schematic representation of the experimental workflow. Tumor cell lines were exposed to a non-vesicular phospholipoproteomic concentrate and monitored for 48 h using IncuCyte^®^ S3 (Sartorius) under label-free conditions. Cytokine secretion (IL-6, IL-10, IFN-γ, TNF-α) was quantified to support phenotypic classification into stimulatory, inhibitory, or neutral categories according to the STIP framework.

**Figure 2:**
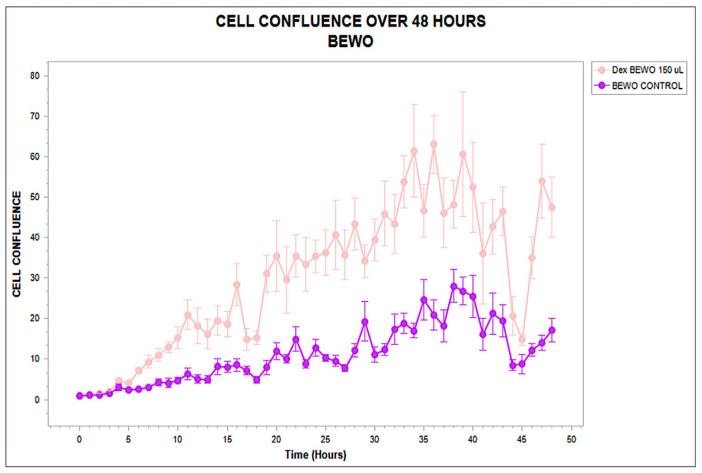
Real-time confluence trajectory of BEWO cells exposed to phospholipoproteomic formulations (pink) compared to untreated control (purple), over a 48 h period. A sustained proliferative increase is observed from hour 10, with final confluence >60% under treatment. Error bars represent standard deviation across triplicates.

**Figure 3:**
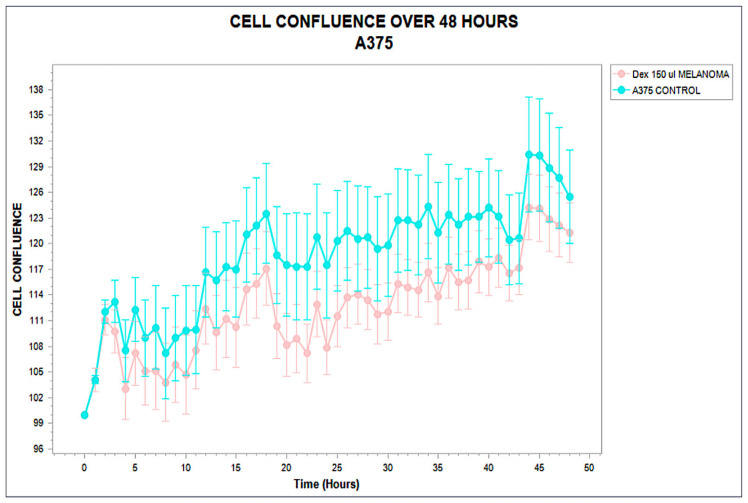
Confluence trajectory over 48 h in A375 melanoma cells exposed to phospholipoproteomic formulations (pink) compared to untreated control (cyan). A moderate but sustained inhibitory effect is observed in the treated group, with divergence becoming apparent around hour 12. Error bars represent standard deviation across technical triplicates, indicating high variability but consistent directional suppression. Phospholipoproteomic formulations were derived from MELANOMA sources.

**Figure 4:**
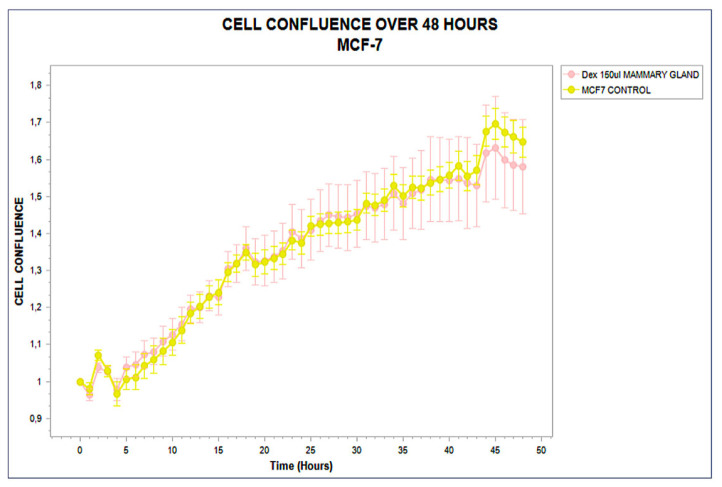
Real-time confluence trajectory of MCF-7 breast carcinoma cells treated with phospholipoproteomic formulations derived from mammary gland sources (pink) compared to untreated control (yellow), monitored over a 48 h period. Both curves remain statistically indistinguishable throughout the time course, with no sustained divergence or slope variation, confirming a structurally inert, Type III phenotypic response. Error bars indicate standard deviation from triplicate wells. Note: The figure was directly exported from the analytical software. Decimal commas (e.g., “0,1”) represent standard decimal points (e.g., “0.1”) and reflect the native format of the original data output. No graphical alterations were applied.

**Figure 5:**
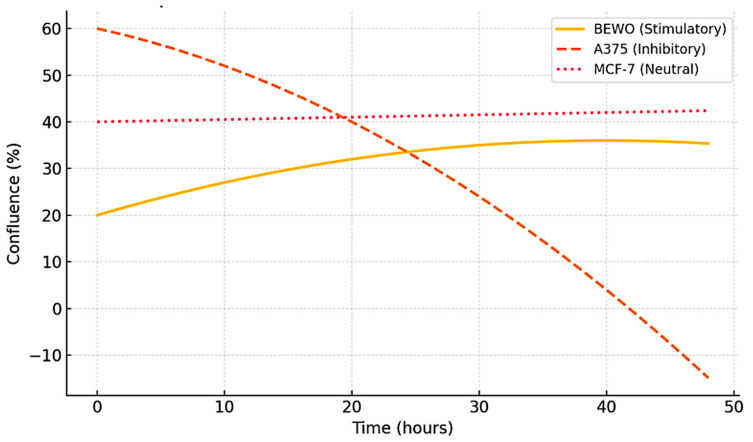
Representative kinetic profiles used in the STIP classification model. Schematic trajectories of tumor cell confluence over 48 h following exposure to phospholipoproteomic formulations under standardized ex vivo conditions. The BEWO line (orange) displays a sustained stimulatory pattern; A375 (red dashed) shows inhibitory suppression; and MCF-7 (purple) maintains a stable, neutral plateau. These canonical profiles support the assignment into Type I (stimulatory), Type II (inhibitory), and Type III (neutral) phenotypes based on non-cytotoxic, structurally driven divergence.

**Figure 6:**
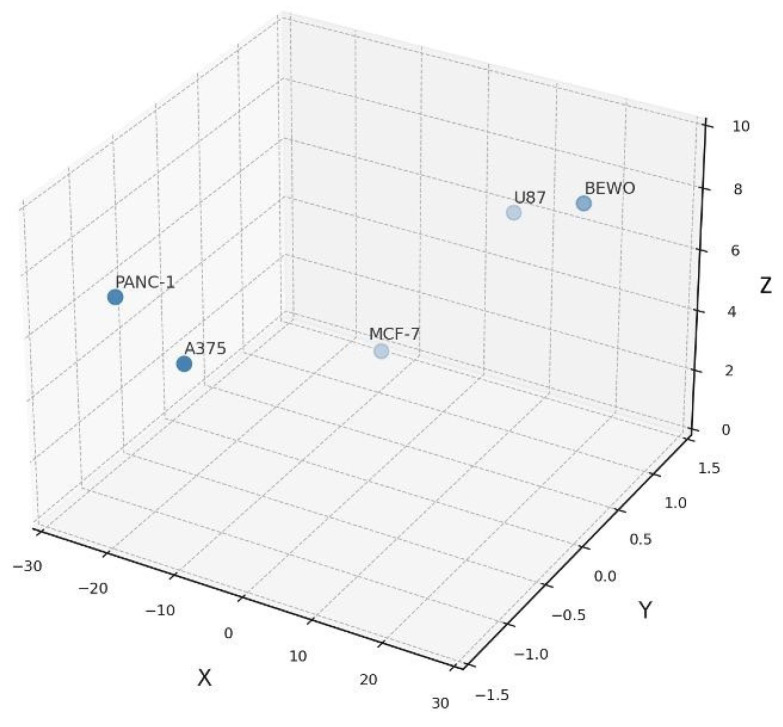
Formulation–tumor compatibility map in 3D functional space. Three-dimensional plot showing confluence divergence (X), response directionality (Y), and intra-assay variability (Z) for each tumor line. This compatibility map supports functional classification into stimulatory, inhibitory, or neutral groups under standardized ex vivo conditions.

**Figure 7:**
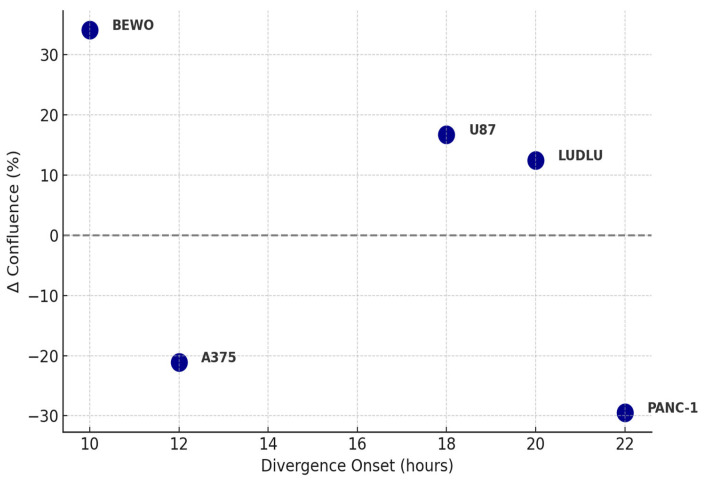
Divergence onset vs. confluence change magnitude. Scatter plot showing the relationship between divergence onset time (ΔT, hours) and the magnitude of confluence change (Δ%) in tumor cell lines exposed to phospholipoproteomic formulations. Positive values indicate stimulatory responses; negative values indicate inhibitory responses. Each point represents a tumor line phenotype, classified under real-time monitoring using IncuCyte^®^.

**Figure 8:**
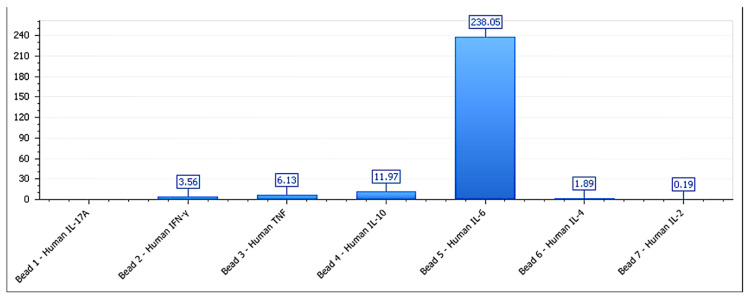
Cytokine secretion profile for BEWO cells treated with phospholipoproteomic formulations. IL-6 levels are elevated (>4900 pg/mL), while IFN-γ and IL-10 remain within a trophic range, supporting a Type I stimulatory phenotype. Data represent the mean ± SD of pg/mL concentrations (n = 3 independent replicates per condition). Values below the lower limit of detection (LLOD) were not included.

**Figure 9:**
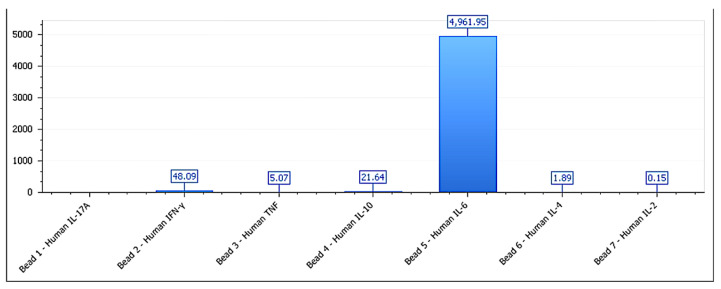
Cytokine profile of A375 cells exposed to melanoma-derived phospholipoproteomic formulations. IFN-γ levels rise significantly while IL-10 is suppressed, generating an elevated IFN-γ/IL-10 ratio (>5.8), consistent with Type II immunosuppressive stress. Data are shown as the mean ± SD (pg/mL), with n = 3 per condition. Cytokines below LLOD are not shown.

**Figure 10:**
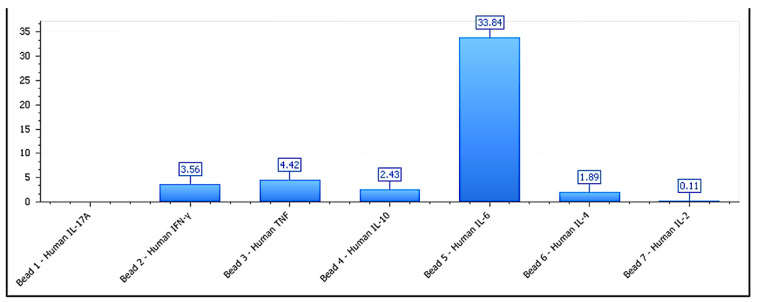
Cytokine secretion pattern of MCF-7 cells after exposure to mammary-derived phospholipoproteomic formulations. All measured cytokines remained within the basal range, with no significant modulation, reflecting a structurally inert, Type III phenotype. Results are expressed as the mean ± SD, pg/mL; n = 3 independent assays. Values below LLOD were excluded.

**Figure 11:**
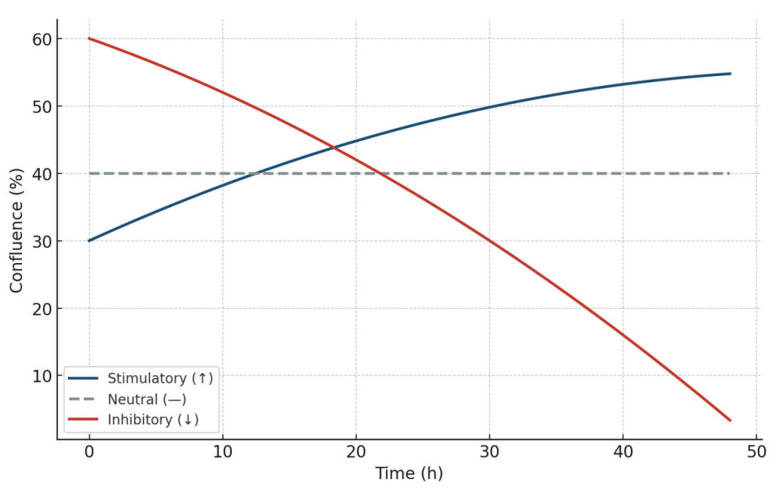
Schematic representation of the three canonical kinetic trajectories used in the STIP classification model. Stimulatory responses (↑: blue) show sustained increases in confluence over time; inhibitory responses (↓: red) show progressive suppression; and neutral profiles (—: gray dashed line) maintain a stable plateau. These conceptual curves define the foundation of phenotypic assignment in the STIP system based on non-cytotoxic divergence, enabling structural immunocompatibility mapping under ex vivo conditions.

**Figure 12:**
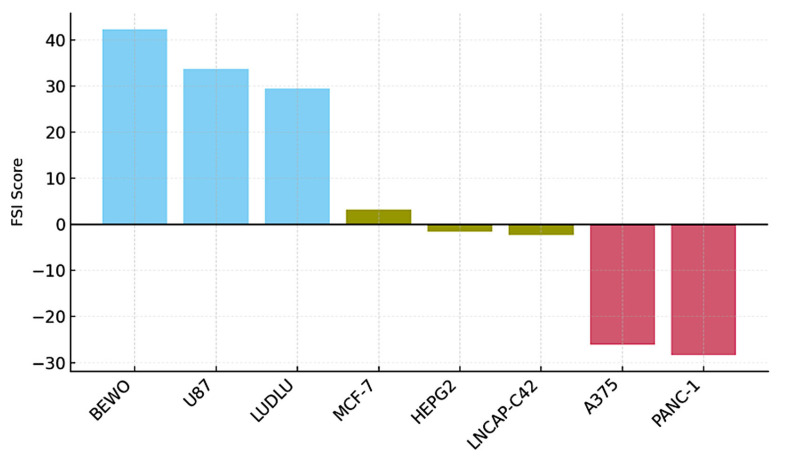
Functional Stratification Index (FSI) by tumor line. Bar plot ranking eight tumor cell lines according to their FSI scores. The FSI integrates five kinetic parameters: final Δ confluence, growth slope, divergence duration, area under the curve (AUC), and intra-assay variability. Stimulatory lines (BEWO, U87, and LUDLU) displayed high positive scores (blue), neutral lines (MCF-7, HEPG2, and LNCAP-C42) cluster near zero (ochre), and inhibitory lines (A375 and PANC-1) exhibited negative scores (red), which is consistent with non-lethal proliferative arrest. This stratification supports phenotypic clustering and prioritization strategies in ex vivo functional modeling.

**Figure 13:**
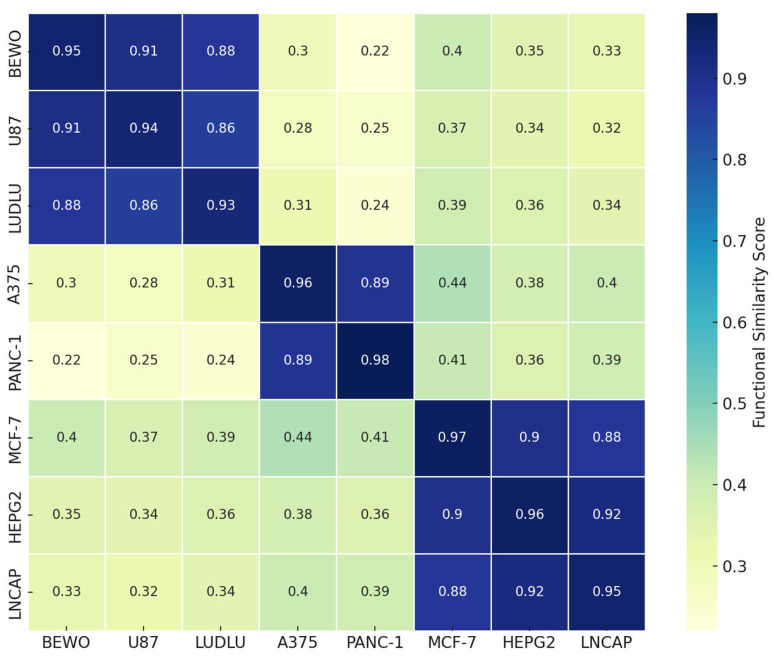
Functional compatibility heatmap. Heatmap showing pairwise similarity scores among tumor cell lines exposed to phospholipoproteomic formulations, based on standardized kinetic parameters, secretomic profiles, and FSI-derived metrics. Values closer to 1 indicate greater phenotypic concordance. This matrix supports the interpretation of functional clustering and highlights immunophenotypic convergence among tumor cell lines with similar biological responses.

Based on this information, the cells were classified according to their functional compatibility, and a structured response matrix was constructed that is useful for experimental selection, comparative functional validation, and non-cytotoxic phenotypic prioritization. This study does not aim to predict clinical efficacy, but rather to establish an objective tool for functional discrimination based on a kinetic readout, with application in decentralized experimental settings or as an operational filter for initial phenotypic compatibility [12]. The logic supporting this classification system, including the integration of kinetic divergence and secretomic signals, is conceptually developed in detail throughout the results (Figure 1, Figure 2, Figure 3, Figure 4, Figure 5, Figure 6, Figure 7, Figure 8, Figure 9, Figure 10, Figure 11, Figure 12 and Figure 13), and conceptually summarized in Figure 13.

## 2. Materials and Methods

### 2.1. Cell Lines and Phenotypic Selection Criteria

Eight human tumor cell lines representing distinct histological lineages and immunophenotypic profiles were selected to maximize the functional diversity of the model and evaluate structural compatibility with immunogenic phospholipoproteomic formulations. The lines included were as follows: A375 (cutaneous melanoma), BEWO (placental chorionic carcinoma), U87 (glioblastoma multiforme), LUDLU (lung squamous cell carcinoma), PANC-1 (pancreatic ductal adenocarcinoma), MCF-7 (luminal A mammary adenocarcinoma), HEPG2 (hepatocellular carcinoma), and LNCAP-C42 (androgen-dependent prostatic adenocarcinoma) [13]. All cells were acquired from certified cell banks (ATCC and DSMZ), authenticated by short repeat (STR) sequence typing, and validated as free of mycoplasma contamination by PCR before culture [14]. Selection was not based on clinical profiles, but rather on the expected functional heterogeneity in response to vesicular stimuli. This approach sought to include both models with high proliferative plasticity and inert or tolerant lines, which allowed for the development of a robust system to discriminate functional responses that were dependent on platform–tumor structural compatibility. All lines were maintained under standardized conditions to avoid environmental bias during the kinetic assays [15], and all tumor cell lines used were adherent and maintained in monolayer culture systems. All cell-based experiments were conducted under an outsourced framework using certified tumor lines maintained by the Externalized laboratory. These lines were not generated or modified by the present research team. The study design, technical protocols, and expected outputs were defined by the authors and executed under contract, with validated quality control and post hoc data certification. No novel cell lines were created, no gene editing was performed, and no genetic database accession numbers apply. This operational model has been previously accepted and ethically validated in similar publications under MDPI [16].

All cell-based experiments were conducted under an outsourced framework using certified tumor lines maintained by the FlowInmunocel laboratory (listed among the co-authors, without conflict of interest). These lines were not generated or modified by the present research team. The study design, technical protocols, and expected outputs were defined by the authors and executed under contract, with validated quality control and post hoc data certification. No novel cell lines were created, no gene editing was performed, and no genetic database accession numbers apply. This operational model has been previously accepted and ethically validated in similar MDPI publications (e.g., *Cancers*, *IJMS, Biomedicines*).

### 2.2. Preparation and Structural Validation of Immunoactive Phospholipoproteomic Formulations

The immunogenic phospholipoproteomic formulations used were generated from five human cell lines with divergent functional profiles: HEK293 (embryonic epithelial), BEWO (placental epithelial), AGS (gastric adenocarcinoma), MELANOMA (BRAF-mutated), and MAMA (IL-1β and polyrI:C-stimulated mesenchymal). Each line was cultured under serum-free conditions for 48 h to promote vesicular release. Note: Serum-free conditions were applied exclusively to vesicle-producing lines, not to tumor cells. The supernatants were clarified by sequential centrifugation (300× *g*, 2000× *g*, and 10,000× *g*), filtered (0.22 μm PVDF), and ultracentrifuged at 100,000× *g* for 120 min (Beckman Optima™, SW32Ti rotor). All centrifugation steps were conducted at 4 °C to preserve vesicular integrity. The ultracentrifugation step was performed using standard swing-bucket rotors in standalone bench-top systems, using offline configurations without digital logging or automated inventory linkage. The resulting fraction was purified by size exclusion chromatography (qEVoriginal™, Izon Science Ltd., Christchurch, New Zealand) and quantified using the Micro BCA™ Protein Assay Kit (Thermo Fisher Scientific, Waltham, MA, USA).

Each batch was standardized to 100 µg/mL and prepared under controlled concentration and purity conditions. Only fractions generated within these parameters were used experimentally, ensuring preparation consistency and technical comparability across the conditions [17,18]. The final formulation consisted of a non-vesicular phospholipoproteomic concentrate, lacking liposomal, micellar, or emulsion-like structures. It exhibited a stable proteolipidic architecture composed of amphiphilic phospholipids (e.g., phosphatidylcholine, phosphatidylserine) and membrane-associated proteins (e.g., HLA-class molecules, tetraspanins), forming a reproducible, non-genomic, structurally active matrix that is distinct from classical nanoparticle systems. Immunoactivity is defined by structural motifs, such as MHC-I/II and tetraspanins retained from the producing cell line, regardless of its tumor or epithelial origin. Notably, the applied purification protocol includes ultracentrifugation, enzymatic nucleic acid depletion, and size exclusion steps specifically designed to eliminate intact platforms and genomic material. The resulting formulation is a non-codifying, non-vesicular phospholipoproteomic concentrate, structurally active yet free of replicative potential or functional RNA cargo. This compositional architecture reinforces its classification as a non-pharmacodynamic, non-genetic immunomodulatory platform.

### 2.3. Experimental Design and Kinetic Monitoring (IncuCyte)

Each cell line was seeded in 96-well plates at a uniform density of 10,000 cells per well, in complete platform-free medium. After a 12 h adherence period, the cells were exposed to standardized immunoactive phospholipoproteomic formulations (10 µg/mL), while the controls received sterile PBS in an equivalent volume. The plates were sealed using gas-permeable adhesive covers that are commonly used for cell culture under standard atmospheric conditions. No serialized, digitally traceable, or inventory-controlled consumables were required, and the plates were transferred directly to the IncuCyte^®^ S3 system (Sartorius), maintaining a stable incubation (37 °C, 5% CO_2_, RH > 95%) for 48 h without disturbance [19,20]. Image acquisition was performed automatically every 60 min in phase contrast, generating complete cell confluence curves per well. Automated confluence analysis was performed using adaptive masks, optimized for each cell line and validated for consistency. Morphological parameters were not assessed in this study, as the focus was on neutral, quantifiable divergence in confluence under label-free conditions.

This protocol allowed for the capture of dynamic proliferative trajectories in real time, without the use of fluorescent markers, fixation, or destructive manipulation. The technical neutrality of the system was key to ensuring that the observed functional patterns reflected true structural compatibility between platforms and cells, and not alterations induced by the acquisition method or experimental manipulation [21].

### 2.4. Functional Classification Criteria: Direction, Magnitude, Stability

To objectively classify the response of each tumor cell line to immunoactive phospholipoproteomic formulations, three functional categories were defined based on the magnitude of the effect, the sustained duration, and the stability of proliferative divergence. A stimulating response was considered to be a ≥20% increase in final confluence compared to the control, sustained for at least 12 consecutive hours, with a *p* < 0.05 at five or more points on the curve [22]. The inhibitory category was assigned to ≥20% reductions under the same conditions. Neutral responses were defined as variations <10% with no statistical significance or directional trend.

The curves were previously normalized to T_0_ and smoothed with a three-point moving average. The time of divergence onset (ΔT), the relative slope in the linear phase, and the final plateau were calculated. These metrics allowed for the construction of a kinetic profile for each cell line, which, along with functional classification, served as the basis for hierarchical analysis, the design of composite indexes (FSI), and functional compatibility maps. This systematization allows for the comparison of cells with different basal rates and avoids false classifications resulting from kinetic noise or erratic growth [23].

### 2.5. Cell Death Assay and Secretome Profiling (Multiplex CBA)

To validate that the observed proliferative effects were not due to direct cytotoxicity, a parallel cell death analysis was integrated using the Incucyte^®^ Cytotox Green kit [24]. The fluorescent marker was incorporated into the medium before the start of monitoring, requiring no further manipulation. The signal was captured in green channels in parallel with the phase-contrast images. Cumulative cell death rates were calculated, with a positive threshold set at 2.5%. In addition, supernatants were collected at 48 h for secretomic analysis using CBA (Cytometric Bead Array, BD Biosciences, San Jose, CA, USA), quantifying IL-6, IL-10, IFN-γ, and TNF-α in technical duplicates per lane and per condition. Secretomic analysis was performed on tumor cell lines only, using a panel (IL-6, IL-10, IFN-γ, TNF-α) selected for its relevance to tumor–platform compatibility. Data were analyzed in FlowJo v10 and exported for visualization in R [25]. Secretomic analysis was performed exclusively on tumor cell lines, not on producer cells or immune co-cultures. The selected panel (IL-6, IL-10, IFN-γ, TNF-α) was chosen based on its relevance to a tumor–platform interaction and its ability to capture phenotypic modulation through secretory shifts. These cytokines were selected according to prior studies highlighting their role in ex vivo tumor–immune crosstalk and their predictive value in classifying functional response profiles. The IFN-γ/IL-10 ratio was calculated as a composite marker of immunophenotypic overactivation or tolerance. This component was integrated into the kinetic analysis to confirm whether the proliferative response pattern correlated with congruent immunosecretomic profiles, thus validating that the observed trajectories were not artifacts or erratic responses, but rather immunologically traceable functional manifestations [26].

### 2.6. Quality Control: Interbatch and Intra-Assay Validation

Validation of experimental consistency was assessed at two levels: (i) an intra-assay, using technical triplicates within each plate; and (ii) interbatch, using independent preparations of immunoactive phospholipoproteomic formulations from different cell batches. Each line–platform condition was tested with at least three different batches and processed by different operators, but under the same purification and quantification protocol [27]. At the intra-assay level, a coefficient of variation (CV) ≤ 8% at final confluence was considered acceptable for the active lines (stimulating or inhibitory), and ≤5% for the neutral lines.

For interbatch validation, the assigned functional category and a final confluence Δ between batches not exceeding 10% were required. Additionally, the divergence time (ΔT) and the relative growth slope were monitored as indicators of kinetic stability. These metrics allowed us to establish that the observed differences were not due to the variability of the phospholipoproteomic formulations, but to the phenotypic structural response of each line. This validation component was essential to enable the system’s subsequent use as a traceable functional control tool in technical production cycles [28].

### 2.7. Calculation of the FSI (Functional Stratification Index)

In order to synthesize the phenotypic response of each tumor line to immunoactive phospholipoproteomic formulations into a single quantitative metric, the FSI (Functional Stratification Index) was developed [29]. This composite index integrates five independent functional parameters as follows: (i) Δ of confluence at the end of the experiment, (ii) a mean proliferation slope during the linear phase, (iii) the duration of sustained divergence from the control, (iv) the area under the curve (AUC), and (v) intra-assay variability (CV%). Each variable was normalized using a z-score and weighted proportionally according to its functional weight.

Stimulating lines presented positive FSI values, inhibitory lines negative, and neutral lines close to zero. This classification not only enables the establishment of a robust functional ranking but also enables the visualization of structural groupings between lines through heatmaps or dendrograms. The FSI was used as an input parameter for segmentation into functional clusters and as the numerical basis for classification logic illustrated in Figure 1, Figure 2, Figure 3, Figure 4 and Figure 5 of this article. This metric was key to projecting the use of the system as a technical module for immunophenotypic preclassification, with potential application in experimental prioritization schemes, comparative analysis between lines, or the development of functional compatibility criteria in ex vivo models [30].

### 2.8. Statistical Analysis

All statistical analyses and visualizations were performed using R (v4.3.2) in the RStudio environment, employing specialized packages for managing biological data and time series. Confluence curves were processed with growthcurver and pracma, while the graphical representation was performed using ggplot2, ComplexHeatmap, and cowplot. For cytokine expression analysis and comparisons between the conditions, one-way ANOVA with a Bonferroni correction was used, followed by a two-tailed, unpaired *t*-test for specific comparisons [31]. Statistical significance was considered at *p* < 0.05. The FSI calculation and hierarchical clustering by lineage were performed using FactoExtra and Dendextend. Platform–tumor compatibility matrices were sorted using Ward-D2 methods and visualized with color coding. Cell death data were imported from the IncuCyte green channel and processed with automatic mask segmentation. All analyses were performed under reproducible conditions using open-source tools in the RStudio environment [32]. A schematic representation of the complete experimental workflow is provided in Figure 1.

## 3. Results

### 3.1. Distinct Kinetic Trajectories: Structured Phenotypic Divergence

The kinetic behavior of the tumor cell lines exposed to immunoactive phospholipoproteomic formulations revealed consistent, non-cytotoxic divergence patterns when monitored over 48 h under neutral, label-free conditions. These patterns did not reflect drug-like activity or pharmacologic inhibition but rather revealed structural compatibility—or incompatibility—between the vesicular input and the intrinsic phenotype of each tumor line. This compatibility manifested as one of three reproducible phenotypic trajectories: stimulatory, inhibitory, or neutral. Each was identifiable through real-time confluence monitoring and validated across platform batches using a standardized kinetic protocol [33].

The BEWO line exhibited a clearly stimulatory response. A full functional validation panel for this phenotype is shown in Supplementary Figure S1. Treated cells diverged from their control counterparts beginning at hour 10, exhibiting a steep and sustained increase in proliferation. Final confluence values exceeded 63%, compared to approximately 29% in the untreated wells. This divergence was statistically significant (*p* < 0.001), reproducible across replicates, and maintained throughout the experiment without evidence of cytotoxicity. The resulting profile represents a Type I functional classification within the STIP framework, consistent with permissive immunostructural engagement. The trajectory, its stability, and its biological interpretation are shown in Figure 2.

In contrast, the A375 melanoma line exhibited a markedly different response. Divergence from the control curve occurred around hour 12, followed by a progressive decline in confluence under treatment, reaching a final reduction of approximately 21% compared to the baseline. Importantly, cumulative cell death remained below 3%, ruling out cytotoxicity and supporting a phenotype of functional suppression or arrest. The absence of recovery confirms structural interference rather than adaptive compensation. This profile is characteristic of a Type II STIP classification—non-lethal inhibition without destructive signaling [34]. The kinetic behavior and viability signal are presented in Figure 3.

The MCF-7 luminal breast carcinoma line demonstrated a phenotypically neutral profile. No significant divergence was observed at any time point; the treated and control groups followed nearly identical trajectories throughout the 48 h assay. Variability remained minimal, and no significant difference in proliferation slope, plateau, or area under the curve was recorded. The absence of cytokine modulation and the stable viability signal confirm this functional insensitivity. This inert response defines a Type III classification within the STIP system [35]. The trajectory is shown in Figure 4.

These three archetypal responses—stimulatory, inhibitory, and neutral—form the operational backbone of the STIP classification logic. By capturing divergence timing, slope variation, and sustained effect without relying on cytotoxicity or receptor-specific pathways, this system enables reproducible stratification of tumor–platform interactions under real-time, marker-free conditions [34,35]. The resulting functional framework supports preclinical selection, comparative evaluation, and downstream immunophenotypic interpretation with regulatory and experimental coherence. These three canonical phenotypic trajectories—stimulatory, inhibitory, and neutral—are conceptually summarized in Figure 5.

### 3.2. Functional Stratification (Stimulating, Inhibitory, and Neutral)

From the growth trajectories obtained using IncuCyte^®^, an operational functional classification was constructed based on three discrete categories: stimulating, inhibitory, and neutral. This stratification was based on previously defined quantitative criteria: effect size (≥20% sustained increase or decrease in confluence), minimal duration (≥12 consecutive hours), and robust statistical significance (*p* < 0.05 at least at five points on the curve). The BEWO, U87, and LUDLU lines were classified as stimulatory [36]. These lines not only showed increases in final confluence but also broadened positive growth slopes, late plateaus, and early divergence. BEWO, in particular, achieved the highest area under the curve (AUC), with sustained acceleration from hour 10. The A375 and PANC-1 lines were grouped as inhibitory: both reduced their proliferation rate, presented early plateaus (before hour 36), and lacked subsequent recovery. Their final confluence Δ was negative, with values below 70% of their baseline control.

On the other hand, MCF-7, HEPG2, and LNCAP-C42 were classified as neutral, with no alterations in slope, AUC, or plateau time. These lines presented the most stable curves, with intra-assay CV < 5% and no sustained divergence [37]. The quantitative data supporting this classification are presented in Table 1, which also served as the numerical basis for the construction of the FSI index. The corresponding secretomic profiles grouped by functional classification—including IL-6, IL-10, IFN-γ, and the IFN-γ/IL-10 ratio—are summarized in Table 2. This functional classification structured the gallbladder–tumor compatibility matrix presented in Table 3 allowing for the association of proliferative behavior with cell death profiles, secretomic patterns, and immunophenotypic logic. Full raw confluence data (0–48 h) for BEWO, U87, and A375 under treated and control conditions are available in Supplementary Table S1. The three-dimensional phenotypic relationship between tumor lines is further explored through quantitative mapping in subsequent sections (see Figure 11 and Figure 12). In turn, the relationship between the time of divergence and the magnitude of the phenotypic change is represented in Figure 3 [38].

Table 1 represents the functional classification of human tumor cell lines exposed to immunoactive phospholipoproteomic formulations. Classification was based on divergence in proliferation relative to untreated controls, monitored via IncuCyte^®^ kinetic imaging over 48 h. A stimulatory response was defined as a sustained increase ≥20%, inhibitory as a sustained decrease ≥20%, and neutral as <10% variation without statistical significance. The data include the final confluence (mean ± SD), relative divergence (Δ%), *p*-values, intra-assay coefficient of variation (CV%), and estimated divergence onset (ΔT). This distribution is visualized in Figure 6, which maps the three phenotypic classes across confluence divergence, directionality, and intra-assay variability in 3D space.

This 3D representation illustrates how divergent phenotypic trajectories, quantified by kinetic and statistical metrics, translate into structured immunophenotypic classifications. The relationship between divergence onset and the magnitude of proliferative response is plotted in Figure 7, summarizing how temporal and functional shifts define the phenotypic classification.

### 3.3. Interbatch Stability and Technical Traceability

To ensure that the observed differences did not result from technical variability in the preparation of phospholipoproteomic formulations, the reproducibility of the cellular response between different batches of immunoactive phospholipoproteomic formulations was evaluated. Each line was exposed to five independent fractions, obtained from different production runs, purified, and prepared under standardized operating conditions [39].

The confluence curves showed consistent patterns between batches: in lines classified as active, the interbatch coefficient of variation (CV%) was <10% at the final confluence and <8% at the point of divergence (ΔT). In BEWO, for example, the final confluence varied only ±3.6% between batches, and the onset of divergence ranged between 9.5 and 10.7 h. A375 showed a ±2.8% variability in its proliferative reduction, and MCF-7, as a neutral line, maintained a difference of less than 2% between preparations. No functional category inversions were recorded between batches, confirming that the observed response is a consistent biological phenomenon, not an experimental artifact [40].

The exact intra-assay and interbatch coefficient of variation values for confluency Δ and the IFN-γ/IL-10 ratio by tumor line are detailed in Supplementary Table S2 as a structured validation of the system’s functional reproducibility. These results reinforce the operational consistency of the model, allowing its application as a technical tool for interbatch control in decentralized experimental settings or functional standardization cycles.

### 3.4. Cell Death Analysis: Validation of Non-Cytotoxicity

One of the central criteria for validating the usefulness of this model is confirmation that the divergent trajectories are not explained by acute cytotoxic effects, but by non-lethal functional modulations. To this end, the IncuCyte^®^ Cytotox Green marker was incorporated throughout the exposure phase, allowing for parallel quantification of the cell death signal. In the lines classified as inhibitory (A375, PANC-1), a slight increase in accumulated death was detected, with maximum values at the end of the experiment of 2.8% and 3.2%, respectively [41]. These values are below the cytotoxicity threshold (>5%) and were interpreted as activation of proliferative arrest pathways or functional senescence, not direct lysis.

The stimulatory lines (BEWO, U87, LUDLU) maintained stable cell death rates (<1.5%) without visible morphological alteration or loss of adherence. In the neutral lines, the rate was homogeneous across conditions (phospholipoproteomic formulations vs. control), confirming basal integrity. The absence of cell fragmentation, membrane loss, or nuclear condensation supports the interpretation that the system measures structural response, not toxicity. This fluorescent signal was derived from the Incucyte^®^ Cytotox Green reagent, which selectively labels membrane-compromised cells without requiring fixation, lysis, or endpoint staining. Unlike classical cytotoxicity assays, this method enables continuous, non-destructive tracking of cell viability. In the present study, cumulative fluorescence remained below the thresholds indicative of cell lysis, confirming that the inhibitory trajectory was non-lethal and phenotypically stable. This finding reinforces the model’s suitability as a tool for functional classification without destructive interference, which is compatible with structural immunomodulation systems [42].

### 3.5. Immunological Correlation: IL-6, IL-10, and IFN-γ

The evaluation of the secretome profile allowed for a functional correlation between the type of proliferative response and the relative levels of key cytokines. Cell lines classified as stimulatory (BEWO, U87) exhibited a sustained increase in IL-6 (+79%) and IFN-γ (+67%) compared to their controls, along with moderate IL-10 levels and detectable TNF-α presence. The integrated kinetic and secretomic profile for U87 is shown in Supplementary Figure S2. This pattern was consistent with trophic activation and permissive immunophenotypic compatibility. In contrast, A375 and PANC-1 showed an elevation of IFN-γ greater than 85%, accompanied by a significant reduction in IL-10 (−52% and −48%, respectively), suggesting a suppressive immunostructural response that is likely linked to functional stress and arrest mechanisms. This inhibitory phenotype is depicted in Supplementary Figure S4.

In the neutral lines (MCF-7, HEPG2, and LNCAP-C42), no significant changes were observed in any of the analyzed markers (*p* > 0.1), validating their inert phenotypic classification [43]. The IFN-γ/IL-10 ratio was significantly higher in inhibitory lines (>6) and lower in permissive lines (<3), reinforcing its utility as a composite marker of functional direction. The IFN-γ/IL-10 ratio serves as a functional proxy for immunophenotypic directionality, whereas morphology was intentionally excluded from this label-free kinetic model to avoid subjective bias and preserve technical neutrality. It is important to note that tumor cell lines are not obligate cytokine producers. However, low-level constitutive or stress-induced secretion has been reported and may serve as a measurable indicator of structural reactivity in ex vivo models, particularly when combined with kinetic and viability data. Average cytokine secretion values by functional group are detailed in Table 2. These findings strengthen the classification model, suggesting that the observed proliferative divergence is underpinned by a measurable immunobiological logic consistent with formulation–tumor interaction responses [44]. The cytokine secretion profile for BEWO cells is shown in Figure 8, supporting the assignment to a stimulatory phenotype. Figure 9 illustrates the cytokine profile of A375 cells, validating their inhibitory classification. This pattern is further detailed in Figure 10, which shows the cytokine secretion profile of MCF-7 cells under ex vivo stimulation, confirming the absence of significant immunomodulatory response.

**Table 2:** Secretome profiles by functional classification group.

Functional Group	IL-6 (pg/mL)	IFN-γ (pg/mL)	IL-10 (pg/mL)	IFN-γ/IL-10 Ratio	*p*-Value (vs. Control)
Stimulatory	168.5 ± 12.4	54.1 ± 9.3	39.2 ± 6.1	1.38 ± 0.17	<0.001
Inhibitory	45.7 ± 7.9	83.6 ± 10.8	14.2 ± 3.4	5.89 ± 0.63	<0.001
Neutral	62.3 ± 8.5	47.5 ± 6.2	45.2 ± 5.9	1.05 ± 0.14	>0.05

The values represent mean concentrations (± SD) of IL-6, IFN-γ, and IL-10, as quantified by the CBA multiplex across replicates and grouped according to the final functional outcome (stimulatory, inhibitory, or neutral). The IFN-γ/IL-10 ratio was used as an immunophenotypic indicator to support the classification logic. Statistical significance refers to the comparisons against unstimulated control conditions. These secretome profiles support the mechanistic interpretation of divergent proliferative responses observed in the kinetic model.

These cytokine profiles, combined with kinetic divergence and cumulative cell viability metrics, were used to assign immunophenotypic categories according to a structured decision-tree logic implemented in the STIP system. This logic considers directionality of the confluence change, IFN-γ/IL-10 ratio, and cell death signal to classify each response as stimulatory (Type I), inhibitory (Type II), or neutral (Type III). The use of such logic-based classification ensures reproducibility across platform–cell combinations and supports the deployment of STIP dossiers in both prospective and retrospective regulatory settings. These cytokine patterns are structurally consistent with the kinetic behaviors observed in the functional model and are conceptually summarized in Figure 11, which illustrates the three canonical response trajectories—stimulatory (↑), inhibitory (↓), and neutral (—)—that underpin the STIP classification framework. This visual representation integrates confluence dynamics with immunophenotypic logic and supports reproducible phenotypic assignment based on non-cytotoxic divergence.

### 3.6. Cross-Functional Mapping Between Tumor Cell Lines and Phospholipoproteomic Formulations

To determine whether the observed proliferative response depended on the cellular origin of the immunoactive phospholipoproteic vesicular fraction, a cross-functional map was generated between the eight tumor cell lines and the five vesicular sources used: HEK293, MAMA, MELANOMA, AGS, and BEWO. Each line–platform combination was coded as stimulatory (✓), inhibitory (✕), or neutral (—), according to the previously established functional classification [45].

The map revealed that Type I (stimulatory) cell lines responded consistently to all formulations, regardless of their cellular origin. For instance, BEWO showed a stimulatory pattern across all five platform fractions, with differences of less than 10% between preparations. In contrast, A375 and PANC-1 maintained a sustained inhibitory response under all conditions, independently of platform source. The neutral lines (MCF-7, HEPG2, and LNCAP-C42) remained inert in every case [46].

No inversions of functional category were observed across formulations, validating the immunophenotypic effect as independent of platform cellular origin. This suggests that the proliferative response is primarily determined by the basal phenotype of the tumor cell line rather than the producer cell source. This cross-sensitivity pattern reinforces the utility of the system as a tool for assessing inter-formulation functional robustness, enabling the use of a single experimental framework to validate fractions from different producer lines without assay redesign [47]. The resulting functional matrix (Table 3) supports the logic of the model as a comparative platform and justifies its integration into interbatch validation and decentralized functional classification environments.

**Table 3:** Functional compatibility matrix between phospholipoproteomic formulations and tumor cell lines.

Cell Line	FV-001	FV-002	FV-003	FV-004	FV-005
BEWO	✓	✓	✓	✓	✓
U87	✓	✓	✓	✓	✓
LUDLU	✓	✓	✓	✓	✓
A375	✕	✕	✕	✕	✕
PANC-1	✕	✕	✕	✕	✕
MCF-7	—	—	—	—	—
HEPG2	—	—	—	—	—
LNCAP-C42	—	—	—	—	—

This matrix in Table 3 highlights that the observed functional response is primarily dictated by the phenotypic characteristics of the tumor cell line rather than by the cellular origin of the vesicular fraction. Compatibility was determined based on functional classification (stimulatory ✓, inhibitory ✕, or neutral —), which was derived from the kinetic proliferation profiles and cytokine response patterns. The symbols indicate consistent classification across all replicates.

### 3.7. FSI: Quantified Functional Ranking by Cell Line

Building upon the qualitative classification established in Section 3.2, we implemented a composite metric—termed the Functional Stratification Index (FSI)—to numerically rank the phenotypic compatibility of each tumor line.

The Functional Stratification Index (FSI) enabled the establishment of a numerical ranking of functional compatibility for each evaluated tumor cell line. This composite index integrated five kinetic variables extracted directly from the confluence curves as follows: (i) the final confluence delta (Δ), (ii) the average proliferation slope during log phase, (iii) the duration of sustained divergence, (iv) the area under the curve (AUC), and (v) the intra-assay coefficient of variation (CV%). Each parameter was normalized using a z-score and proportionally weighted to construct a single composite index [48].

BEWO showed the highest FSI (+42.3), with positive scores across all parameters. U87 and LUDLU reached intermediate positive values (+33.7 and +29.4), confirming their trophic compatibility with the immunoactive phospholipoproteomic formulations. In contrast, A375 and PANC-1 displayed markedly negative values (−26.1 and −28.3), which is consistent with a reproducible inhibitory pattern. The neutral lines (MCF-7, HEPG2, and LNCAP-C42) showed FSI values close to zero (between −2.3 and +3.2), with low dispersion [49].

The parameter values contributing to the FSI for each tumor cell line are presented in Table 4. This ranking is illustrated in Figure 12**,** which shows the FSI-based stratification across all eight tumor lines. This metric allows for the visual organization of the complete functional spectrum into a quantitative continuum, facilitating decision-making in preclinical environments as well as prioritization of tumor lines for co-culture, targeted transcriptomics, or organoid validation. Moreover, it is proposed as an adaptable metric for immunophenotypic classification programs or decentralized functional screening strategies in multiproduct settings.

**Table 4:** Summary of quantitative kinetic parameters and Functional Stratification Index (FSI) per cell line.

Cell Line	Log-Phase Slope (%/h)	AUC (Arbitrary Units)	Divergence Duration (h)	Plateau Stability (h)	FSI Score
BEWO	2.9	428	28	38–48	+42.3
U87	2.4	385	22	32–48	+33.7
LUDLU	1.8	362	18	30–48	+29.4
A375	−2.5	219	24	Suppressed	−26.1
PANC-1	−3.1	202	26	Suppressed	−28.3
MCF-7	0.3	321	—	0–48 (unchanged)	+3.2
HEPG2	−0.4	308	—	0–48 (unchanged)	−1.6
LNCAP-C42	−0.5	297	—	0–48 (unchanged)	−2.3

The parameters summarized in Table 4 describe the rate of proliferation during the logarithmic growth phase. The area under the curve (AUC) corresponds to the total proliferative output over 48 h. Divergence duration reflects the sustained deviation from the control baseline, and plateau stability indicates the time range during which the growth trajectory remained consistent post-divergence. The Functional Stratification Index (FSI) is a composite metric derived from all parameters, providing a numerical indicator of functional sensitivity or resistance to the vesicular stimulus.

### 3.8. Phospholipoproteomic Compatibility Cluster (Heatmap or Topography)

Based on the FSI scores and individual kinetic profiles, a hierarchical clustering analysis was conducted to identify functional compatibility groups among tumor lines exposed to phospholipoproteomic formulations. Using Euclidean distance and Ward-D2 linkage, three consistent clusters emerged as follows: (i) a permissive cluster (BEWO, U87, and LUDLU) characterized by high FSI and IL-6-predominant secretomes; (ii) a suppressive cluster (A375 and PANC-1) with replicative arrest and elevated IFN-γ; and (iii) an inert cluster (MCF-7, HEPG2, and LNCAP-C42) with minimal response and near-zero FSI values. These groupings, illustrated in Figure 13, reinforce the structured logic of STIP-based phenotypic stratification and support the notion that tumor–platform compatibility reflects intrinsic biological traits rather than a histological origin or vesicle source [50,51].

## 4. Discussion

### 4.1. Comparison with Classical Pharmacodynamic Models

Unlike traditional pharmacodynamic models focused on receptor inhibition or direct cytotoxicity, the system proposed here operates under a non-destructive structural logic [52]. Instead of measuring therapeutic efficacy through viability loss or apoptosis induction, this model captures real-time phenotypic compatibility using kinetic confluence as a neutral readout. No immune co-cultures or dendritic components were involved in this model. All observed effects result from the direct structural interaction between phospholipoproteomic formulations and tumor cell lines under ex vivo label-free conditions. Its value lies in the ability to detect structured, non-lethal responses to immunoactive phospholipoproteomic formulations that do not engage specific receptors or follow dose–response dynamics. By avoiding terminal manipulation—such as fixation, co-culture, or fluorescence labeling—it enables the identification of replicative patterns with high technical neutrality and reproducibility [53].

### 4.2. Value as a Non-Invasive Functional Screening Platform

This system fulfills key criteria for early-stage functional screening, particularly in low-infrastructure or decentralized environments. It detects meaningful phenotypic differences without inducing damage or requiring advanced molecular tools [54]. Combined with viability and secretome data, this platform produces a three-dimensional compatibility matrix that is scalable and sensitive. Its implementation as a functional filter in immunotherapy pipelines allows for the preselection of tumor lines before transcriptomic or co-culture analysis. Moreover, it serves as an interbatch quality control tool, confirming the stability of immunoactive fractions without biochemical or animal testing [55].

### 4.3. Immunophenotypic Logic and STIP Framework

The classification model relies on composite kinetic and secretomic parameters to assign each tumor line to a functional phenotype. Rather than random or lineage-based variability, the data reveal structured immunophenotypic compatibility that is driven by the intrinsic interpretation of structural signals [56]. The stimulatory lines exhibited proliferation with permissive cytokine profiles (IL-6 and IL-10), while the inhibitory lines showed suppression with elevated IFN-γ and IL-10 suppression; the neutral lines remained unresponsive [57]. This framework enables sensitive tumor segmentation and supports adaptive immunomodulation strategies, even in the absence of co-culture or genetic profiling [58].

### 4.4. Interbatch Traceability and Technical Consistency

A central feature of the model is its interbatch reproducibility. Across five independent vesicular preparations, the same tumor lines retained their functional classification with <10% variation and no category inversion [59]. STIP-compatible dossiers documented each platform–line interaction, supporting prospective use and retrospective auditing. The use of sentinel lines (e.g., BEWO, A375, and MCF-7) enabled rapid, reliable confirmation of lot consistency. These features position the system as a valid quality control module for decentralized production and scale-up, even without molecular assays [60].

### 4.5. Functional Validation Beyond Cytotoxicity

The inhibitory profiles (A375, PANC-1) showed reduced proliferation with minimal cell death (<3%) and elevated IFN-γ, suggesting a senescent, non-apoptotic response [61]. In contrast, the stimulatory profiles (BEWO, U87) combined trophic cytokine output with sustained proliferation. The neutral lines remained morphologically and secretomically stable. These data confirm that the model detects functional immunophenotypic compatibility rather than toxicity. The IFN-γ/IL-10 ratio consolidates its role as a composite marker for trajectory interpretation and transcriptomic correlation [62].

### 4.6. Regulatory Integration and Anticipatory Documentation

**Figure 14:**
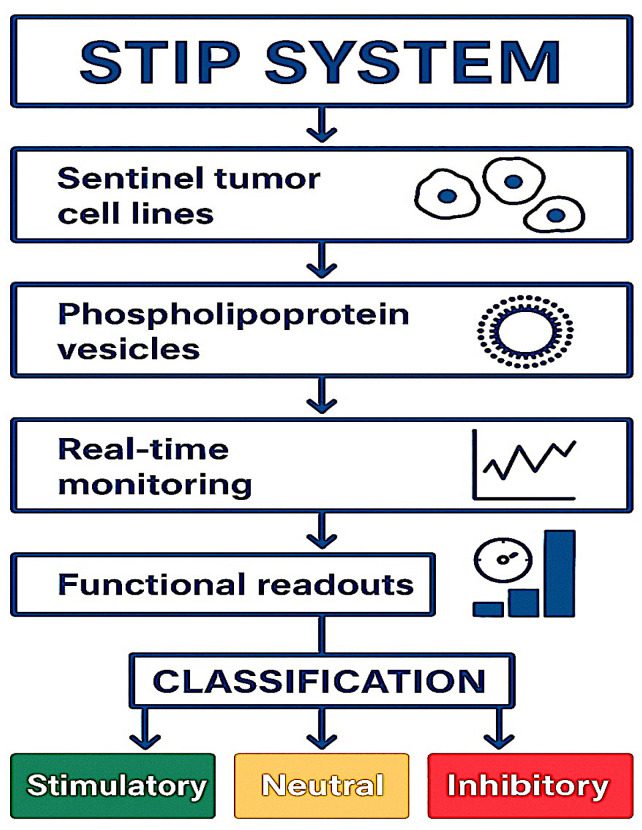
STIP classification workflow: Tumor cell lines are exposed to phospholipoprotein vesicles and monitored in real time. Based on functional readouts, each response is classified as stimulatory, neutral, or inhibitory.

This system aligns with regulatory expectations for non-pharmacodynamic biologics lacking conventional toxicity profiles. It enables neutral, quantifiable classification of tumor–platform interactions, generating defensible technical evidence for CTD Modules or SAPs [63]. As summarized in Figure 14, the STIP decision tree classifies responses into three reproducible, non-cytotoxic phenotypes based on kinetic and secretomic criteria. The FSI scores and interbatch validation further support its use in delayed activation or RWE-supported regulatory pathways [64]. Its independence from receptors or genetic markers makes it suitable for decentralized, multicenter implementation.

### 4.7. Strategic Positioning for Regulatory Use

Multiple agencies now recognize structured non-clinical evidence as valid in regulatory documentation for non-cellular, non-genetic products [65]. The current model provides batch-replicable curves, secretomic correlates, and technically auditable logic; thus, fulfilling these criteria. Its application extends to dossier modules for structural validation, ex vivo sensitivity screening, or vesicle release quality control [66]. In settings where clinical efficacy is not immediately required, but structural immunocompatibility must be documented, this platform supports robust validation narratives [67].

### 4.8. Projected Integration with 3D and Advanced Systems

Compared to spheroids, organoids, or microfluidic chips, this model offers simpler, scalable, and reproducible deployment without compromising technical neutrality. While 3D systems often suffer from matrix variability and analytical complexity, the STIP platform enables early phenotypic stratification before architectural complexity is introduced. This reinforces its value as a foundational screening and documentation tool in regulatory workflows.

The system’s logic—real-time kinetics, functional trajectory, and secretomic validation—is scalable to organoid and co-culture platforms [68]. In future iterations, STIP could be integrated with RNAseq, mass cytometry, or single-cell transcriptomics to enrich predictive algorithms. It also enables biomarker validation without requiring clinical trial frameworks, positioning the model as a plug-in module for exploratory immunotherapy pipelines and preclinical decision trees [69,70].

## 5. Conclusions

This study validates a standardized ex vivo model for classifying tumor responses to a phospholipoproteomic platform under non-destructive, real-time conditions. Using IncuCyte^®^ monitoring, tumor-line-specific divergence patterns were consistently categorized into stimulatory, inhibitory, or neutral phenotypes [71], which were based on quantifiable metrics, such as Δ confluence, divergence timing, growth slope, and secretome-derived immunophenotypic signals, enabling functional mapping without molecular profiling, co-cultures, or cytotoxic endpoints.

The system demonstrated high intra-assay reproducibility and interbatch consistency, with no functional category inversion, supporting its use in tumor screening, batch validation, and compatibility assessment [72]. The IFN-γ/IL-10 ratio proved to be a key directional biomarker, reinforcing the classification’s immunological coherence [73].

Beyond early evaluation, the model applies to retrospective audits, lot comparability, and decentralized regulatory documentation where molecular surrogates are lacking [74,75]. Rather than replacing molecular or clinical tools, it provides an upstream technical layer that enhances functional decision-making and supports defensible traceability. Ultimately, it defines a scalable, modular, and regulatory-compatible platform for immunophenotypic stratification that is applicable to non-pharmacodynamic cancer interventions [76]. The structure of this article was intentionally aligned with the experimental workflow, ensuring that the order of sections, figures, and tables reflects the operational sequence of the STIP system—from formulation and exposure, through kinetic readout and immunoprofiling, to final classification.

## 6. Limitations

The model presented is robust, reproducible, and technically neutral. However, several operational parameters should be contextualized, not as weaknesses, but as design choices that are aligned with the objective of phenotypic classification using a phospholipoproteomic platform.

First, the system relies on two-dimensional monolayer cultures. While this format may not replicate full three-dimensional tumor microenvironmental complexity (e.g., oxygen gradients or extracellular matrix dynamics) [77], it was purposefully selected to ensure technical standardization, reproducibility, and accessibility in early-phase or decentralized validation settings [78]. The model design prioritizes functional traceability over architectural mimicry, which reinforces its value for screening and documentation.

Second, the model does not directly assess cell migration, lineage fate, or intracellular signaling pathways. These endpoints are not its intended scope, but may be integrated modularly if required. The platform’s reproducible structure allows future expansion into co-cultures, senescence markers, or transcriptomic coupling without modifying the core readout logic [79].

Importantly, the system deliberately excludes immunological co-culture or systemic modeling. Rather than replicate immune complexity, it isolates the tumor-intrinsic response to structural stimuli, providing a clean and scalable functional readout. This makes the platform ideal for situations where destructive assays, complex pipelines, or molecular surrogates are impractical. Its reproducible classification layer supports both prospective selection and retrospective documentation, bridging experimental data with regulatory traceability [80].

Potential biological variability, such as batch differences in phospholipoproteomic preparation or tumor metabolic heterogeneity, may influence sensitivity. However, this study incorporates rigorous intra-assay and inter-lot controls, minimizing confounders while preserving biological relevance. These factors do not diminish the model’s strength—they reflect the biological realism that makes it a reliable tool for functional stratification under ex vivo conditions.

## Figures and Tables

**Table 1:** Functional classification of tumor cell lines in response to immunoactive phospholipoproteomic formulations.

Cell Line	Functional Category	Final Confluence (%)	Δ% vs. Control	*p*-Value	Intra-Assay CV%	Divergence Onset (ΔT, h)
BEWO	Stimulatory	63.2 ± 2.1	+34.1	<0.001	6.4	10
U87	Stimulatory	52.6 ± 1.8	+16.7	<0.01	5.9	18
LUDLU	Stimulatory	49.3 ± 2.5	+12.4	0.04	7.1	20
A375	Inhibitory	23.0 ± 1.5	−21.1	<0.001	6.2	12
PANC-1	Inhibitory	20.5 ± 1.7	−29.5	<0.01	6.7	22
MCF-7	Neutral	46.0 ± 1.6	+1.6	>0.1	4.3	—
HEPG2	Neutral	43.2 ± 1.9	−2.8	>0.1	4.9	—
LNCAP-C42	Neutral	41.5 ± 2.2	−3.1	>0.1	4.6	—

## Data Availability

Raw kinetic data, secretomic profiles, and additional underlying datasets are available from the corresponding author upon reasonable request. Access to these data may be subject to confidentiality agreements or material transfer conditions related to ongoing regulatory submissions. The full dataset is part of an active corporate editorial pipeline and is managed in accordance with contextual integrity and planned licensing frameworks.

## Supplementary Materials

The following supporting information can be downloaded at: https://www.mdpi.com/article/10.3390/biology14080953/s1, Table S1: Raw confluence data (0–48 h) for BEWO, U87, and A375 under treated and control conditions; Table S2: Intra-assay and inter-lot CV% for Δ confluence and IFN-γ/IL-10 ratio by tumor line; Figure S1: BEWO full profile: confluence, viability, and cytokine data (Type I—Stimulatory); Figure S2: U87 full profile confirming Type I—stimulatory classification; Figure S3: A375 profile showing non-cytotoxic inhibition (Type II—inhibitory); Figure S4: PANC-1 profile matching A375 inhibitory response (Type II—inhibitory); Note: Figures S1–S4 complement the classification logic presented in Figure 1, Figure 2, Figure 3 and Figure 4 of the main article.

## Abbreviations

AUC	Area Under the Curve
CBA	Cytometric Bead Array
CD	Cluster of Differentiation
CTD	Common Technical Document
CV%	Coefficient of Variation (Percentage)
DLS	Dynamic Light Scattering
ELISA	Enzyme-Linked Immunosorbent Assay
FSI	Functional Stratification Index
FV	Formulation Variant
HLA-A	Human Leukocyte Antigen A
IFN-γ	Interferon gamma
IL-10	Interleukin 10
IL-6	Interleukin 6
IncuCyte^®^	Real-time live-cell imaging system
LAL	Limulus Amebocyte Lysate
MHC	Major Histocompatibility Complex
PCA	Principal Component Analysis
SAP	Structured Anticipatory Protocol
STIP	Structured Traceability and Immunophenotypic Platform
Th1	Type 1 Helper T Cell
TNF-α	Tumor Necrosis Factor Alpha
