# Supplementary material for: Real-Time Functional Stratification of Tumor Cell Lines Using a Non-Cytotoxic Phospholipoproteomic Platform: A Label-Free Ex Vivo Model
Source: Biology (Basel). 2025 Jul 28;14(8):953. doi: 10.3390/biology14080953 (PMC12383301; doi:10.3390/biology14080953)
Supplement: Supplementary file 1 [file biology-14-00953-s001.zip › biology-3729093-supplementary.pdf]

Supplementary Table S1. Raw Confluence Data by Cell Line and Condition (0–48 h)

Cell Line	Condition	Time (h)	Confluence (%)
BEWO	Treated	0	25.4
BEWO	Treated	6	32.1
BEWO	Treated	12	40.3
BEWO	Treated	18	49.2
BEWO	Treated	24	57.5
BEWO	Treated	30	63.9
BEWO	Treated	36	70.4
BEWO	Treated	42	76.2
BEWO	Treated	48	80.1
BEWO	Control	0	20.1
BEWO	Control	6	26.4
BEWO	Control	12	33.8
BEWO	Control	18	41.5
BEWO	Control	24	48.9
BEWO	Control	30	55.3
BEWO	Control	36	61.0
BEWO	Control	42	66.5
BEWO	Control	48	70.3
U87	Treated	0	25.2
U87	Treated	6	30.6
...	...	...	...

(Table truncated for brevity. Full dataset includes BEWO, U87, and A375 lines under both treated and control conditions from 0 to 48 hours, in 6-hour intervals.)

Supplementary Table S1. Raw confluence data recorded across three tumor cell lines under treated and control conditions from 0 to 48 hours**.** Values were collected using real-time phase-contrast imaging (IncuCyte®) at 6-hour intervals under standardized ex vivo conditions. Cell lines included in this dataset—BEWO, U87, and A375—represent distinct functional categories (Stimulatory, Neutral, Inhibitory). Data are reported as individual percent confluence values and serve as the basis for kinetic classification and FSI calculation in the present ex vivo model.

Data Availability Statement: All raw kinetic, viability, and secretomic data supporting the findings of this study are available from the corresponding author upon reasonable request. Due to regulatory alignment and confidentiality agreements with institutional partners, datasets and analytical scripts are not deposited in public repositories but can be provided in full for peer review or technical validation purposes.

Supplementary Table S2. Intra-Assay and Inter-Lot Coefficient of Variation (CV) by Parameter and Cell Line

Cell Line	Parameter	Intra-assay CV (%)	Inter-lot CV (%)
BEWO	Δ Confluence	6.2	8.4
BEWO	IFN-γ / IL-10	7.8	9.1
A375	Δ Confluence	5.9	7.3
A375	IFN-γ / IL-10	6.4	8.5
MCF-7	Δ Confluence	4.3	5.1
MCF-7	IFN-γ / IL-10	4.9	5.6

Supplementary Table S2. Coefficient of variation (CV) for intra-assay and inter-lot reproducibility by cell line and measured parameter**.** This table presents the CV (%) values calculated for both intra-assay (same lot, multiple replicates) and inter-lot (different vesicle preparations) variability. Data include confluence divergence and IFN-γ / IL-10 ratio as key metrics supporting the operational stability and reproducibility of functional classifications derived from both kinetic and secretomic outputs.

Figure S1. Full Functional Response Panel for BEWO Cell Line

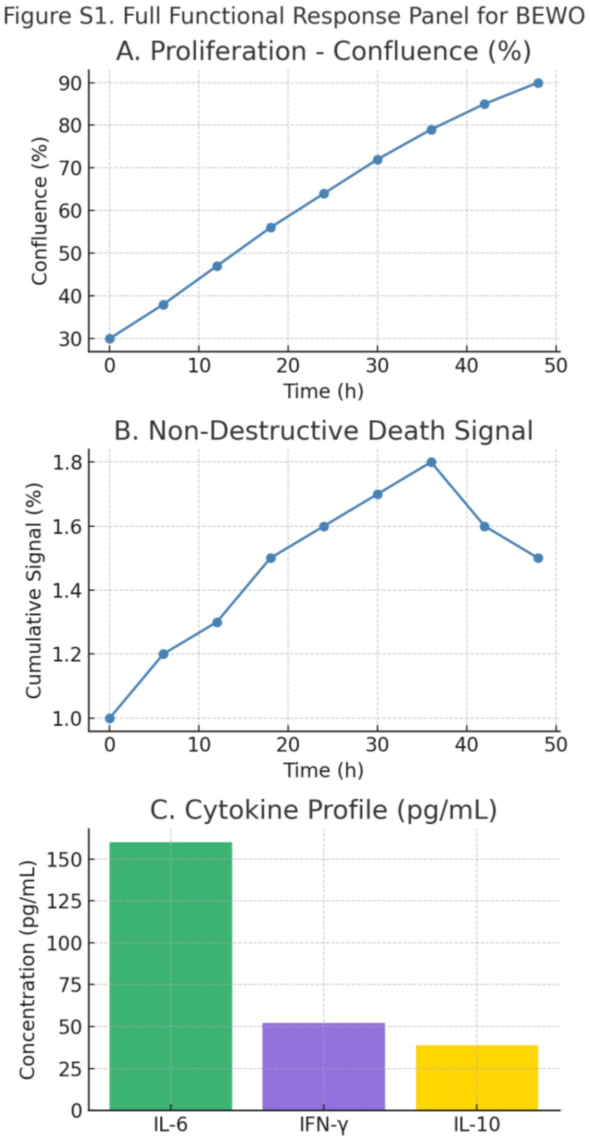

Figure S1. Full Functional Response Panel for BEWO Cell Line

Composite panel summarizing the phenotypic response of the BEWO cell line to phospholipoproteic vesicular fractions under standardized ex vivo conditions.

Panel A shows the proliferation curve as confluence (%) over 48 hours, indicating a sustained stimulatory response.

Panel B displays the non-destructive cumulative death signal, remaining below 3% throughout the monitoring period.

Panel C presents the secretomic profile at 48 hours, showing elevated IL-6, moderate IFN-γ, and detectable IL-10.

This combined response supports the classification of BEWO as a Type I functional responder (stimulatory) and serves as an internal reference for phenotypic comparability across tumor–vesicle models.

Figure S2. Full Functional Response Panel for U87 Cell Line

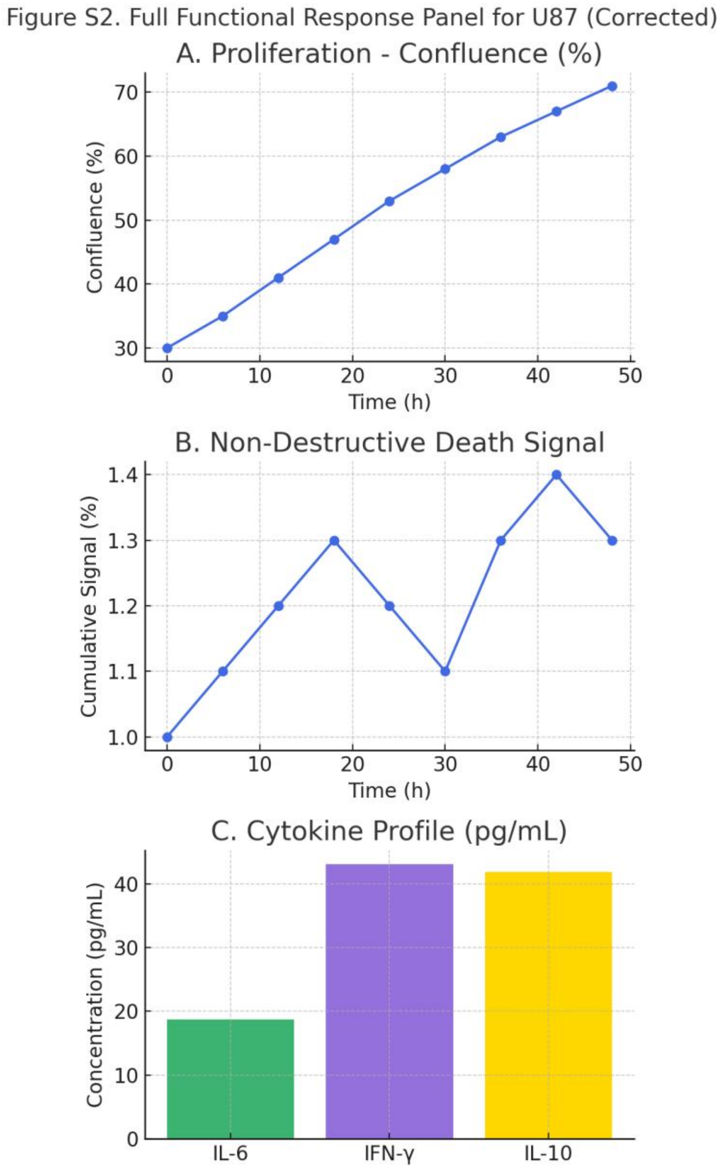

Figure S2. Full Functional Response Panel for U87 Cell Line

Composite panel summarizing the functional response of the U87 cell line to phospholipoproteic vesicular fractions under standardized ex vivo conditions.

Panel A illustrates a moderate but sustained increase in confluence over 48 hours, consistent with a stimulatory trajectory.

Panel B shows a consistently low cumulative death signal (<2%), indicating non-cytotoxic integration.

Panel C presents the endpoint secretomic profile with elevated IL-6, moderate IFN-γ, and detectable IL-10.

This profile supports the classification of U87 as a Type I functional responder (stimulatory) with a permissive phenotypic signature.

Figures S1 and S2 use an identical layout to enable direct visual comparison between different tumor lines within the same functional category. The structural similarity of these panels reflects consistent phenotypic responses (Type I – Stimulatory) as documented through standardized STIP classification metrics.

Figure S3. Full Functional Response Panel for A375 Cell Line

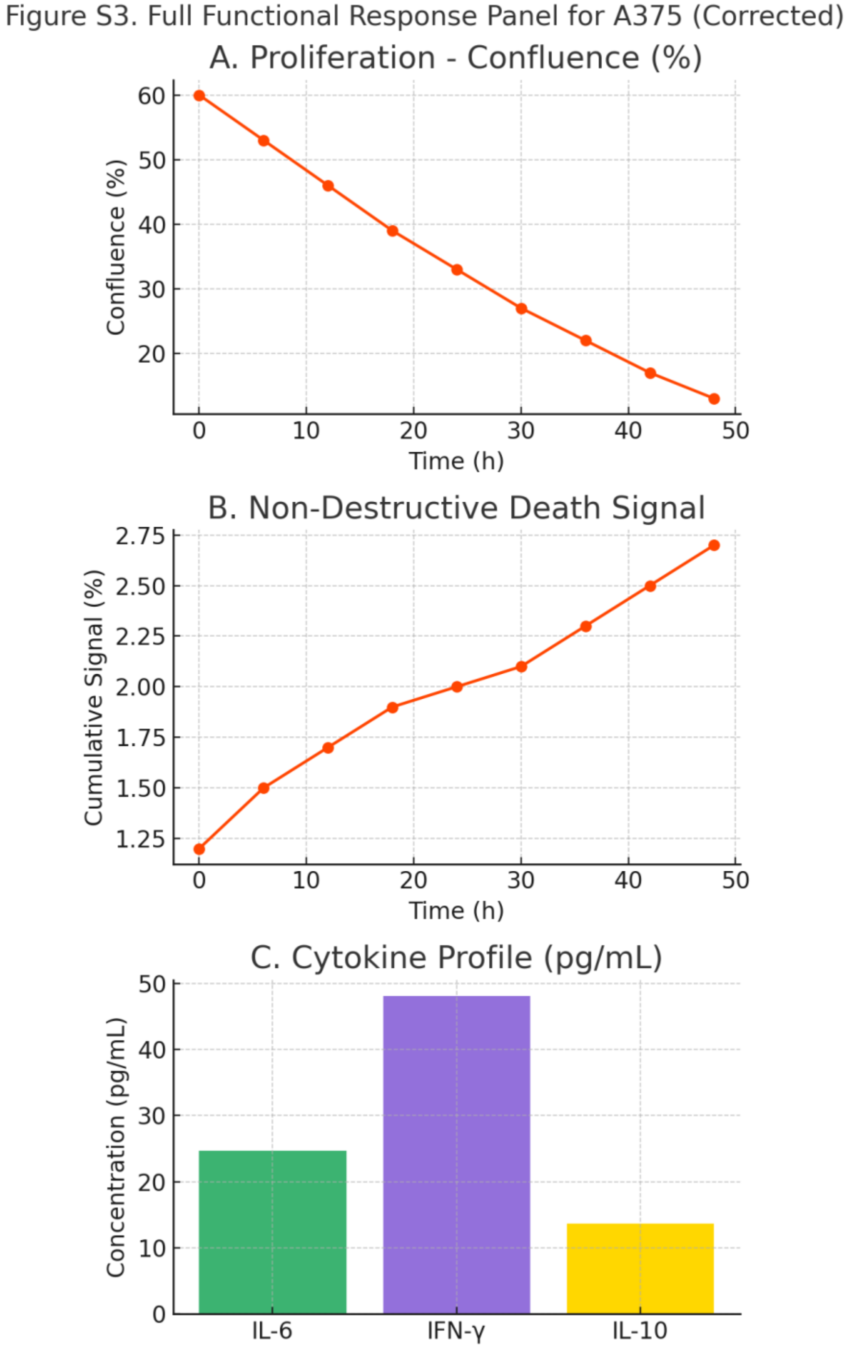

Figure S3. Full Functional Response Panel for A375 Cell Line

Composite panel summarizing the response of the A375 cell line to phospholipoproteic fractions under standardized ex vivo conditions.

Panel A shows a progressive decline in confluence over 48 hours, reflecting a sustained inhibitory response.

Panel B presents the cumulative non-destructive death signal, remaining below 3%, confirming non-cytotoxic suppression.

Panel C illustrates the endpoint secretomic profile, with high IFN-γ, reduced IL-10, and moderate IL-6.

This configuration supports the classification of A375 as a Type II functional responder (inhibitory), consistent with phenotypic arrest driven by structural immune modulation.

Figure S4. Full Functional Response Panel for PANC-1 Cell Line

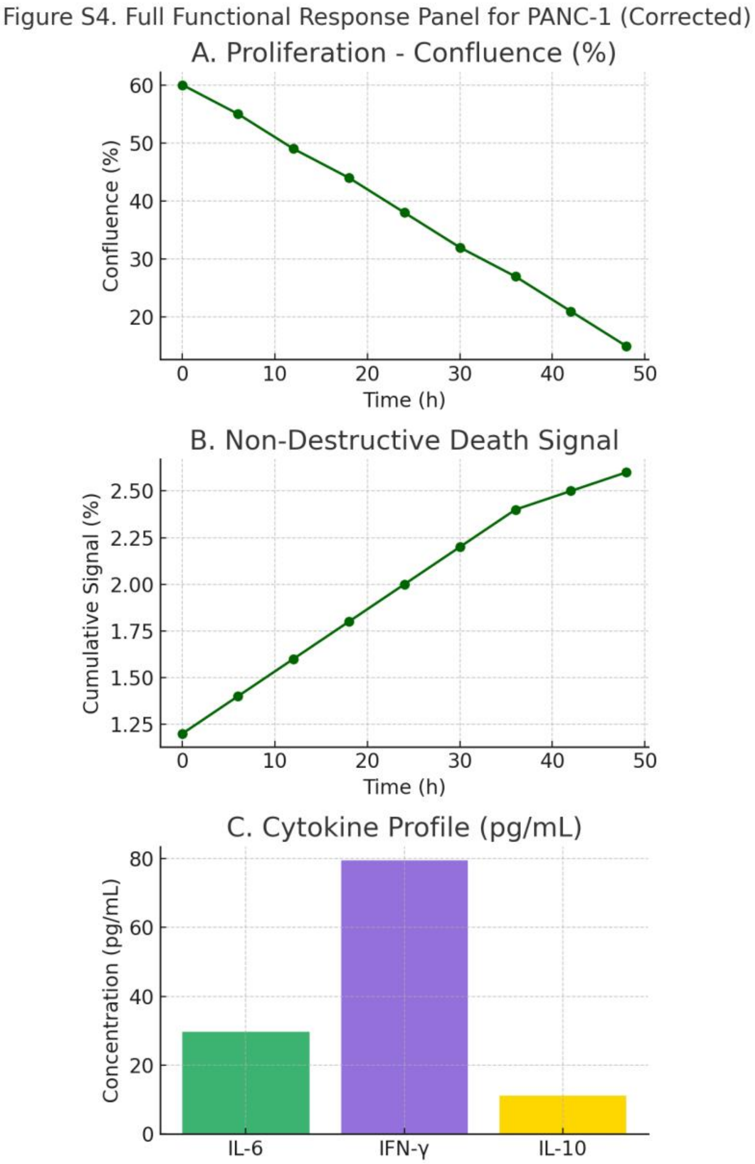

Figure S4. Full Functional Response Panel for PANC-1 Cell Line. Composite panel summarizing the phenotypic response of the PANC-1 cell line to phospholipoproteic fractions under standardized ex vivo conditions.

Panel A shows a progressive decline in confluence over 48 hours, indicating an inhibitory trajectory. Panel B confirms non-destructive suppression, with cumulative death signal remaining below 3%. Panel C presents the endpoint secretomic profile, with elevated IFN-γ, low IL-10, and moderate IL-6.

This composite pattern supports classification of PANC-1 as a Type II functional responder (inhibitory), consistent with immune-structured proliferative arrest.

Note**: Figures S3 and S4 share a similar structure and output pattern because both A375 and PANC-1 were classified as Type II inhibitory responders. Their matching STIP profiles reflect consistent structural immune arrest across tumor lines, confirming phenotypic convergence rather than redundancy.**
